# Supplementary figures and images for: Congruence between preferred and actual place of death and its association with quality of death and dying in advanced cancer patients: A nationwide survey in Japan
Source: PLoS One. 2025 Jul 7;20(7):e0320541. doi: 10.1371/journal.pone.0320541 (PMC12233252; doi:10.1371/journal.pone.0320541)

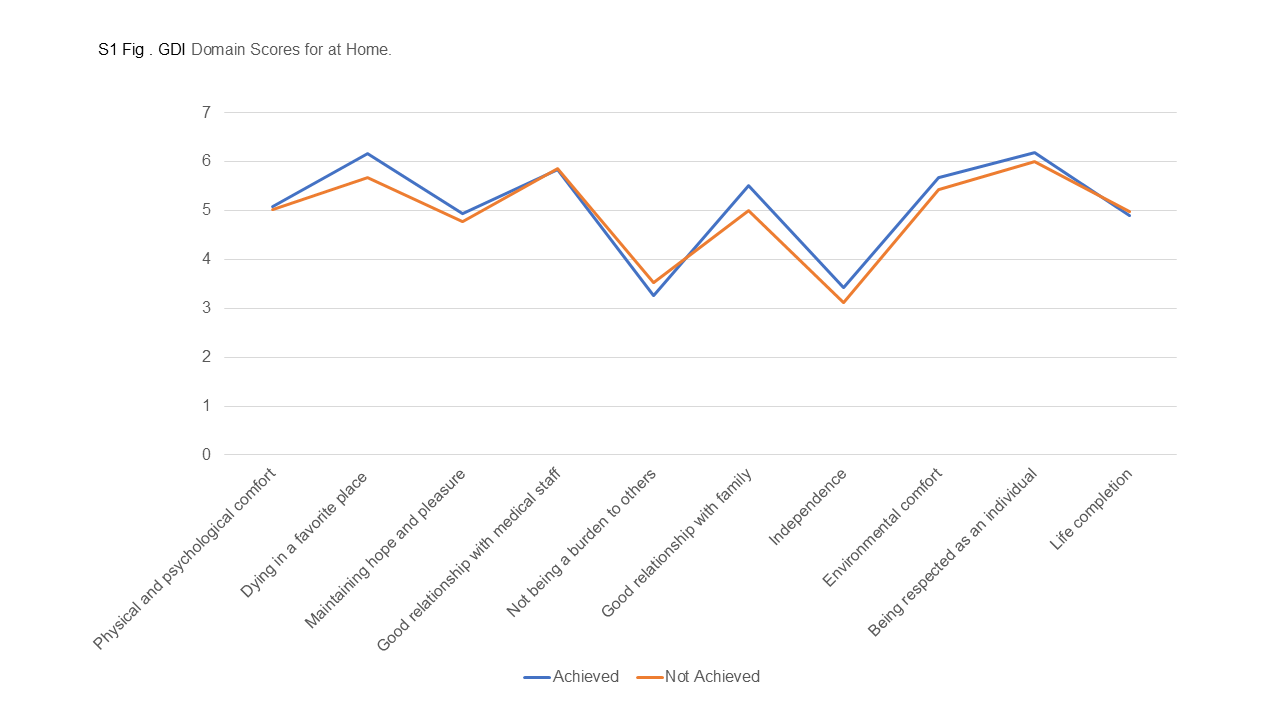

Supplement: S1 Fig — (TIF) [file pone.0320541.s001.tif]

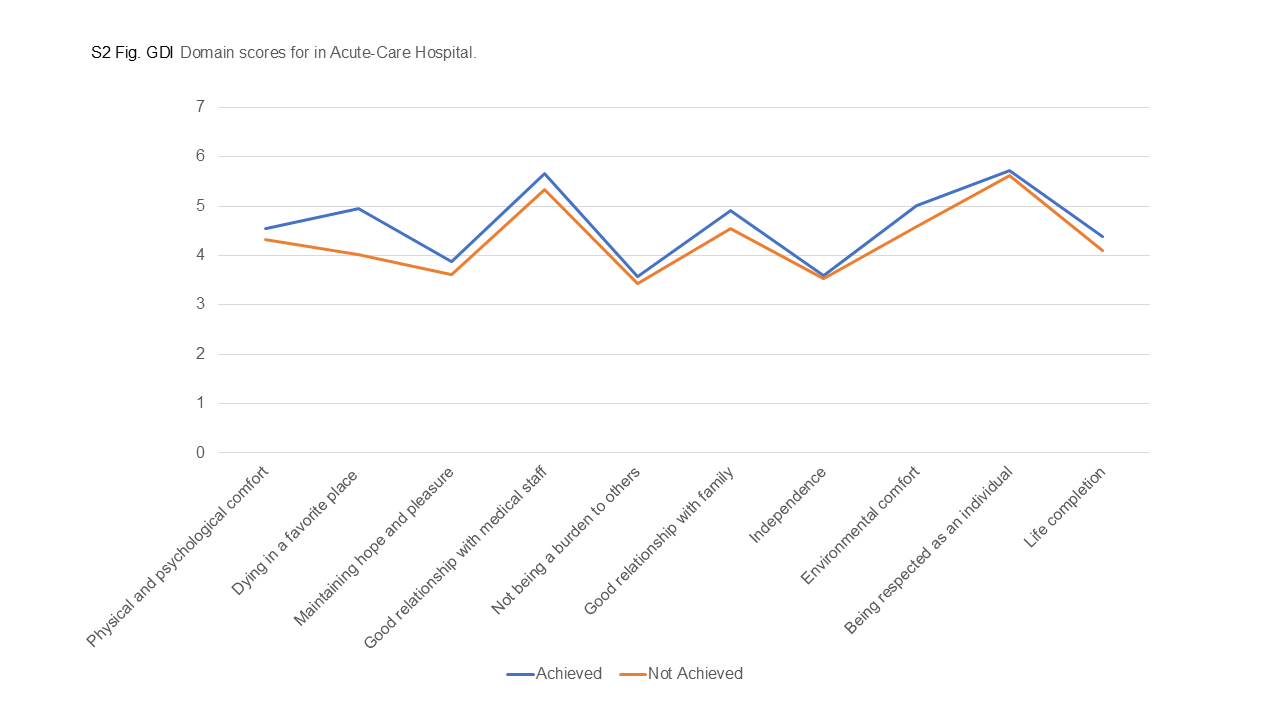

Supplement: S2 Fig — (TIF) [file pone.0320541.s002.tif]

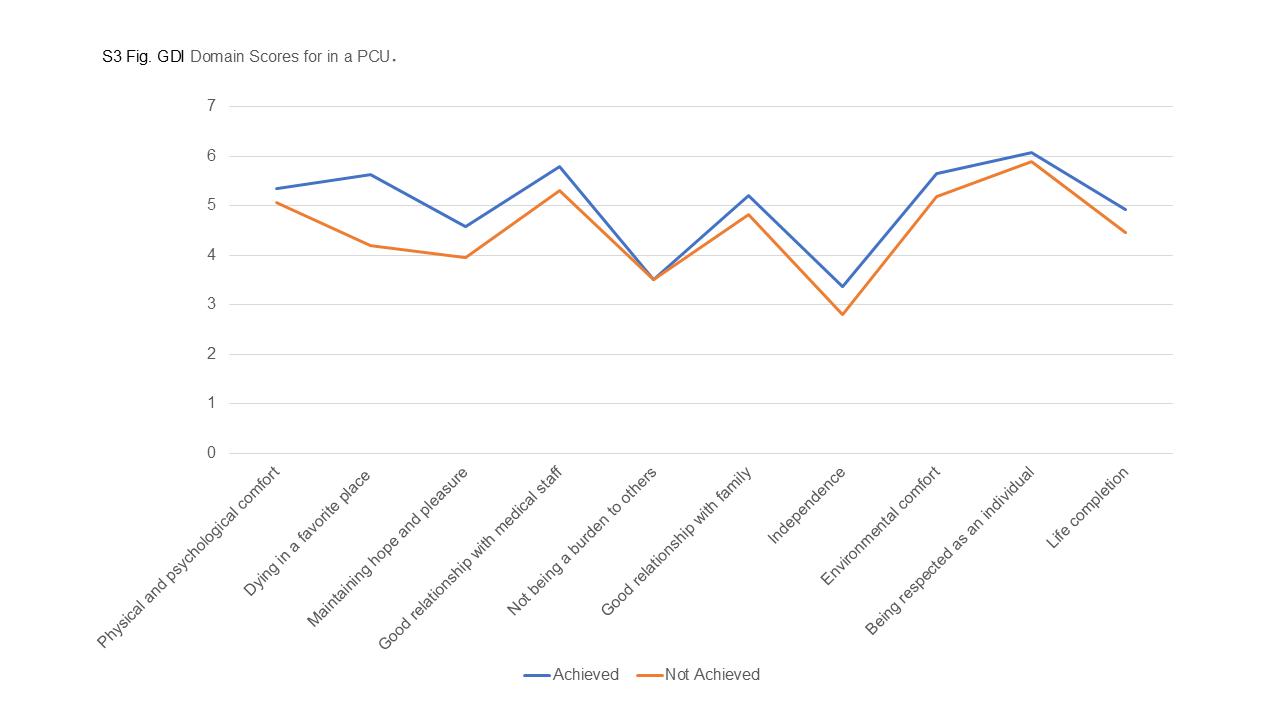

Supplement: S3 Fig — (TIF) [file pone.0320541.s003.tif]
