# Supplementary material for: Congruence between preferred and actual place of death and its association with quality of death and dying in advanced cancer patients: A nationwide survey in Japan
Source: PLoS One. 2025 Jul 7;20(7):e0320541. doi: 10.1371/journal.pone.0320541 (PMC12233252; doi:10.1371/journal.pone.0320541)
Supplement: Attachment — Submitted filename: POD_reviewer_2025_april8,2025.docx [file pone.0320541.s005.docx]

**Congruence between preferred and actual place of death and its association with**

**patients’ quality of death and dying: A nationwide survey in Japan**

| **Topics** | **Comments** |
| --- | --- |
| Abstract | 1.The abstract should specify the number of participants for data analysis, providing a clear understanding of the study's scope and scale.  2.Could you please clarify what the survey score refers to? |
| Introduction | 1. Before Line 54, please clarify why the congruence between the preferred and actual place of death is important and how it relates to achieving a good quality of death. Additionally, consider providing a brief definition or explanation of what constitutes a good quality of death.  2. Line 58-59, please make sure that what the dependent variable in this study was, and it should be added to the hypothesis. Is the dependent variable the good death or end-of-life quality? Do these have the same meaning? More importantly, the instrument that measured a dependent variable for what purpose: good death or end-of-life quality? Please clarify. |
| Purpose and aim | In the abstract, two purposes of the study are stated. However, at the end of the introduction, it appears that the study has only one aim: 'to evaluate the association between this congruence and patients’ quality of death and dying, as perceived by their family caregivers.' Please ensure consistency between the abstract and the introduction. |
| Methods | **Data set**  I just want to confirm that I understand the question about the preferred place of death correctly: this information was reported by the bereaved family caregiver, not obtained directly from the patient while they were still alive—in other words, the patient was not asked where they preferred to die. However, there is descriptive data listed under the 'preferred place of death' heading that appears to reflect the patient's preferences, which seems inconsistent. Could you please clarify?  **Questionnaires**  Please clarify the source of the data on the actual place of death. Was this information reported by the family caregiver? If so, please make this clear in the methods section.  **Analysis**  1.How do you define congruence between the preferred and actual place of death, and how do you test or calculate this congruence?  2.Please revise this statement for understanding "which pair of groups the differences lay?".  3. What p-value threshold is used to determine statistical significance? Please clearly state this in your analysis (e.g., p < .05 or p < .01), as it will help readers better interpret the p-values presented in the table alongside the 95% confidence intervals.  4. In the multiple regression analysis, please clearly specify which factors were included in the model to predict the GDI (Good Death Index). Also, please clarify the meaning of the "place of death" variable—does it refer to the preferred place of death, the actual place of death, or the congruence between the two?  5. From my understanding of your results, it seems you examined two types of congruence: (1) between the patient’s preferred and actual place of death, and (2) between the caregiver’s preferred and the patient’s actual place of death. Please clarify which type of congruence was used in the analysis and reported in the results. Additionally, indicate which was used to examine factors associated with the GDI. The analysis and results sections were somewhat confusing in this regard. |
| Results | **Table 1**  1.Number of people should describe in paragraph instead of adding it within the Table 1.  2.Check categories of “Gender” both in patients and family caregiver characteristics: Male or Female?  3. For family caregiver characteristics, preferred home as the place of death for their loved one data should be added.  4.Should add * in each p-value that is significant, for example, p<.0001*  5.Check grammar in this sentence: “Total of some items does not add to 100% owing to missing data”.  **Preferred place of death**  Can you please clarify how you test/calculate the congruence between the preferred and actual place of death in the data analysis?  **Table 2.**  1. Please check if you spell “maintaining” correctly throughout the manuscript and figure.  2.Please check the percent of the congruence rate in lines 174-176 to be aligned with the percent that is shown in the percent of actual place of death (achieved) in Table1. And please clarify how you calculate the congruence rate in the analysis.  **Congruence between preferred and actual place of death and its association with quality of death and dying:** Which congruence measure was used in the analysis of factors associated with this outcome? Was it the congruence between the patient’s preferred and actual place of death, or the congruence between the bereaved caregiver’s preferred place of death for their loved one and the actual place of death? In the discussion section, the study refers to the congruence between the patient’s preferred and actual place of death. Please ensure consistency throughout the manuscript regarding which type of congruence was used in the analysis.  Lines 222-226: Please rewrite these sentences for better clarity and review which factors show significant differences in Table 3.  In Table 3, which coefficient is used for the regression between the standardized and unstandardized coefficients? Please clarify. |
| Discussion | 1.Please consider discussing which factors affect the congruence between the preferred and actual place of death.  2.I don't know whether this study measures quality of life. Be mindful not to overstate the findings of the study. Please align the discussion with the results (the dependent variable is the quality of dying).  3. Are you considering future research aimed at increasing the congruence rate between patients’ preferred and actual place of death? |
| Conclusion | It is a concise conclusion. |
