# Supplementary material for: Congruence between preferred and actual place of death and its association with quality of death and dying in advanced cancer patients: A nationwide survey in Japan
Source: PLoS One. 2025 Jul 7;20(7):e0320541. doi: 10.1371/journal.pone.0320541 (PMC12233252; doi:10.1371/journal.pone.0320541)
Supplement: Attachment — Submitted filename: Response to Reviewers.docx [file pone.0320541.s007.docx]

Gursharan K Singh, PhD

Academic Editor

*PLOS ONE*

04 June 2025

Dear Dr Singh,

Re: Manuscript reference No. PONE-D-25-05638

Please find attached a revised version of our manuscript “Congruence between preferred and actual place of death and its association with advanced cancer patients’ quality of death and dying: A nationwide survey in Japan,” which we would like to resubmit for publication as an Original Article in *PLOS ONE*.

Your comments and those of the reviewers were highly insightful and enabled us to greatly improve the quality of our manuscript. In the following pages, please find our point-by-point responses to each of the reviewers’ comments as well as your own comments.

Revisions in the text are shown using yellow highlight for additions and strikethrough font for deletions. In accordance with a suggestion from Reviewer 1, we have *stated the focus on patients with cancer in the title and included references to recent studies*. We hope that the revisions in the manuscript and our accompanying responses will be sufficient to make our manuscript suitable for publication in *PLOS ONE*.

We look forward to hearing from you at your earliest convenience.

Yours sincerely,

Dr Mariko Shutoh

Minato Home Care Clinic, Chuo-ku, Tokyo, Japan

Ph: +81 358590812

Fax: +81 358590822

E-mail: shut44105m@gmail.com

**Responses to the comments of Reviewer #1**

1. This study only focuses on patients with cancer, right? If so then this should be made clear in the title and the aim, and a motivation to why the study is limited to only patients with cancer and not other chronic illness with potential palliative care would be good.

*Response:*

*Line 2: The title has been changed to make it clear that the study relates to cancer patients.*

*Lines 45–55: We have added text to clarify why the study was limited to cancer patients.*

2. The introduction is easy to follow and contains adequate parts, and leads onto a rational and the research questions. The introduction could be developed and present more about common preferences for care at the end of life and place of death as well as quality indicators and qualitative care at the end of life. Pls see some recommended references below.

There are more recent studies on place of death as well as preferences thereof, the introduction and discussion could be strengthened with more reference and description of an international context.
Example studies place of death Europe:
Öhlén, J., Stina, N., Anneli, O., Stefan, N., Hanna, G., Johan, F. C., & Cecilia, L. (2025). Influence of palliative care policy on place of death for people with different cancer types: a nationwide’register study. PloS one, 20(3), e0320086.
Cohen, J., Pivodic, L., Miccinesi, G., Onwuteaka-Philipsen, B. D., Naylor, W. A., Wilson, D. M., ... & Deliens, L. (2015). International study of the place of death of people with cancer: a population-level comparison of 14 countries across 4 continents using death certificate data. British journal of cancer, 113(9), 1397-1404.
O'Sullivan, A., Larsdotter, C., Sawatzky, R., Alvariza, A., Imberg, H., Cohen, J., & Öhlén, J. (2024). Place of care and death preferences among recently bereaved family members: a cross-sectional survey. BMJ Supportive & Palliative Care, 14(e3), e2904-e2913.
Cross, S. H., & Warraich, H. J. (2019). Changes in the place of death in the United States. New England Journal of Medicine, 381(24), 2369-2370.

*Response:* *Lines 58–66: In the revised introduction, we have added information about common preferences for care at the end of life and place of death, as well as quality indicators and qualitative care at the end of life.*

3. Data collection, how long after the death did the bereaved family members respond to the survey, was there any reasoning about when to send the survey invite? I see this is mentioned only as a limitation with regard to recall bias, a descroiåiption under methods would be helpful too. This is important for several reasons, firstly not too soon for ethical reasons of potentially upsetting the family members, secondly not too close to the yearly anniversary of the death for the same reason, thirdly the question of recall bias, if too long time has passed since the death.

*Response:* *Lines 100-102: In the revised methods section, we have included information about when the survey was sent out (and why).*

4. Setting, a setting description would be helpful describing the type of care available and the end of life in Japan and how healthcare is organised in Japan, if it is universal or not. There is a really high proportion of the patients that had died in PCU compared to results from place of death other countries, see examples of references for Europe below.

*Response: Lines 45–54: An explanation of the current state of palliative care in Japan has been added to the revised introduction.*

*We have also added a limitation to the discussion (lines 324-326), as follows: “First, the participants were recruited from facilities that were members of HPCJ, which has a high percentage of PCU members. The proportion of patients who died in PCUs in the present study was therefore relatively high, and the findings may not wholly represent the extent of palliative care services in Japan**.”*

5. Previous research has shown how the preference may change closer to death and how the last days or week may be spend in the preferred place, but that the actual death may take place in hospital or hospice care. Could you differentiate the place for care the last few days and the place of death or did family members only give one response for last few days of care and death together?

*Response: In contrast to western countries, where the place of death and the place of end-of-life care before death are considered separate, the two are not considered separate in Japan.*

*One of the exclusion criteria of this study was that the patient received palliative care for less than 3 days. Therefore, patients who received care for 4 days or more were included in this study, and the place of care received within 4 days of death matched the place of death.*

6.  Is there any information about the non-responders and if they differ from the responders?

*Response: Unfortunately, there is no information on non-responders and no analysis has been performed.*

7. Intervention of palliative care team lower % than the 80% dying in PCU for achieved place, doesn’t PCU mean an automatic intervention of a palliative care team?

*Response: In this study, the term “palliative care teams” was used to refer to palliative care teams operating in general hospitals. To clarify this point, "Intervention of a palliative care team" has been revised to "Intervention of* *a hospital-based palliative care team."*

8. Table 1, under relationship total there is no % presented for son, daughter in law etc…The % for education total most be wrong, does not add up to 100?

*Response: Thank you for pointing this out; we have added the correct numbers to revised Table 1. The education percentages do not always add up to 100% because of missing data.*

9. Row 185: maybe state the place in connection with the % for this sentence “The congruence rates between caregivers’ preferred and actual place of death for their loved ones were 32.1%, 93.2%, and 38.7% for home, PCU, and acute-care hospital, respectively”.

*Response:* *We have corrected this point in the revised manuscript.*

10. It would be good if you could clearer describe that the preferred place for care and death is the patient’s preference as reported by the family member as a proxy for the patient, but also that you asked about the family member’s preferences for where the patient should be care for and place of death, if I have understood this correctly as described under the results? This could be made clearer under data collection.

*Response:* *The following items have been added to the Questionnaire (lines 127–128): “In which place of care did you wish [the patient] to spend his or her last days?”*

11. The differences between patients’ and family members’ preferences. Congruences between family members’ preferences and actual place of death?

*Response: Thank you for pointing this out. We have added “Congruences between family members’ preferences and actual place of death” to revised Table 1 to show the differences between patients’ and family members’ preferences. These are also mentioned in the revised discussion.*

12. In the future extend research to include other diagnoses with potential palliative care needs than only patients with cancer?

*Response: Although we do not currently have any specific plans, we would like to conduct research into congruence between the preferred and actual place of death in non-cancer patients in the future.*

**Responses to the comments of Reviewer #2**

1. The abstract should specify the number of participants for data analysis, providing a clear understanding of the study's scope and scale.

*Response: We have added this information to the revised abstract (line 31).*

2. Could you please clarify what the survey score refers to?

*Response: We have corrected “survey scores” to “Good Death Inventory scores” (line 34).*

3. Before Line 54, please clarify why the congruence between the preferred and actual place of death is important and how it relates to achieving a good quality of death. Additionally, consider providing a brief definition or explanation of what constitutes a good quality of death.

*Response:* *We have added the requested information to the revised manuscript (lines 58-61).*

4. Line 58-59, please make sure that what the dependent variable in this study was, and it should be added to the hypothesis. Is the dependent variable the good death or end-of-life quality? Do these have the same meaning? More importantly, the instrument that measured a dependent variable for what purpose: good death or end-of-life quality? Please clarify.

*Response: The* *dependent variable was the quality of death and dying (measured using the Good Death Inventory score). We had considered that “the quality of death and dying” and “the quality of end-of-life” were interchangeable; however, we accept that this may be confusing to readers. We have therefore standardized this idea to “the quality of death and dying” throughout the revised manuscript.*

5. In the abstract, two purposes of the study are stated. However, at the end of the introduction, it appears that the study has only one aim: 'to evaluate the association between this congruence and patients’ quality of death and dying, as perceived by their family caregivers.' Please ensure consistency between the abstract and the introduction.

*Response:* *Thank you; this point has been unified, as follows:*

*Abstract: “This study aimed to evaluate the association between the congruence between a patient’s preferred and actual place of death and their quality of death and dying, as perceived by their family caregivers.”*

*Introduction: “The aim of this study was to evaluate the association between this congruence and patients’ quality of death and dying, as perceived by their family caregivers.”*

6. I just want to confirm that I understand the question about the preferred place of death correctly: this information was reported by the bereaved family caregiver, not obtained directly from the patient while they were still alive—in other words, the patient was not asked where they preferred to die. However, there is descriptive data listed under the 'preferred place of death' heading that appears to reflect the patient's preferences, which seems inconsistent. Could you please clarify?

*Response: Congruence between the preferred and actual place of death was retrospectively assessed by asking family caregivers, “In which place of care did he or she wish to spend his or her last days?” and* *“In which place of care did you wish [the patient] to spend his or her last days?”*

*The question "In which place of care did you wish [the patient] to spend his or her last days?" was not included in the original manuscript, so we have added it to the revised manuscript for clarity (lines 127-128).*

7. Please clarify the source of the data on the actual place of death. Was this information reported by the family caregiver? If so, please make this clear in the methods section.

*Response:* *To clarify this process, we have added the following text to the revised methods (lines 134-137): “The questionnaires were sent by the medical institutions that participated in the study and certified each patient's death. By linking the data obtained from medical institutions with the returned questionnaires, we were able to identify the place of death.”*

8. How do you define congruence between the preferred and actual place of death, and how do

you test or calculate this congruence?

*Response: For each case, the patient's preferred place of death (as reported by the caregiver) was compared with the actual place of death to determine whether there was congruence (i.e., to determine whether the two places were the same). We then calculated the number of patients for whom there was congruence.*

9. Please revise this statement for understanding "which pair of groups the differences lay?".

*Response:* *Thank you for this comment. For clarity, we have changed the text to "which groups the differences lay." (Line 170).*

*As shown in S1 Table, ANOVA models were used to examine the relationships among the “achieved group,” “not-achieved group,” “no preference,” and “preference unknown.” When significant differences were observed, a post hoc Dunnett test was used to investigate between which groups the differences lay.*

10. What p-value threshold is used to determine statistical significance? Please clearly state this in your analysis (e.g., p < .05 or p < .01), as it will help readers better interpret the p-values presented in the table alongside the 95% confidence intervals.

*Response:* *In response to your comment, the following text has been added to the revised manuscript:* *“Significance was set at P < 0.05.”*

11. In the multiple regression analysis, please clearly specify which factors were included in the model to predict the GDI (Good Death Index). Also, please clarify the meaning of the "place of death" variable—does it refer to the preferred place of death, the actual place of death, or the congruence between the two?

*Response: The multiple regression analysis was performed using all items listed in Table 3 as predictors of the GDI.*

*Actual place of death: The actual place where the patient died.*

*Congruence between preferred and actual place of death: The patients died at the patients’ preferred place (as reported by the caregiver).*

*Congruence between family caregivers’ preferred and actual place of death: The patients died at the family caregivers’ preferred place.*

*We have also added the item “Congruence between family caregivers’ preferred and actual place of death” and conducted the multiple regression analysis again.*

12. From my understanding of your results, it seems you examined two types of congruence: (1) between the patient’s preferred and actual place of death, and (2) between the caregiver’s preferred and the patient’s actual place of death. Please clarify which type of congruence was used in the analysis and reported in the results. Additionally, indicate which was used to examine factors associated with the GDI. The analysis and results sections were somewhat confusing in this regard.

*Response: The section on “Congruence between preferred and actual place of death” in the “Questionnaires” has been revised to make it easier for readers to understand (line 127-128).*

*As you have stated, we calculated both the congruence between the patient’s preferred place of death and the actual place of death, and the congruence between the caregiver's preferred place of death and the actual place of death as reported by caregivers. Nonetheless, the main purpose of our paper was to clarify the association between the congruence between the patient’s preferred place of death and actual place of death and the GDI scores.*

13. Number of people should describe in paragraph instead of adding it within the Table 1.

*Response: We have added the number of people in the paragraph.*

14. Check categories of “Gender” both in patients and family caregiver characteristics: Male or Female?

*Response: This has been corrected to male in* *Table 1*.

15. For family caregiver characteristics, preferred home as the place of death for their loved one data should be added.

*Response:* *We added an item regarding “Congruence between preferred and actual place of death” to the caregiver characteristics in Table 1.*

16. Should add * in each p-value that is significant, for example, p<.0001*

*Response: Thank you; we have made the suggested change.*

17. Check grammar in this sentence: “Total of some items does not add to 100% owing to missing data”.

*Response: Thank you; we have changed this text to “Totals of some items do not add to 100% owing to missing data.”*

18. Can you please clarify how you test/calculate the congruence between the preferred and actual place of death in the data analysis?

*Response: For each case, the patient's preferred place of death reported by the caregiver was compared with the actual place of death to determine whether there was a match, and the number of patients for whom there was a match was calculated.*

19. Please check if you spell “maintaining” correctly throughout the manuscript and figure.

*Response: Thank you; we have made the suggested correction.*

20. Please check the percent of the congruence rate in lines 174-176 to be aligned with the percent that is shown in the percent of actual place of death (achieved) in Table1. And please clarify how you calculate the congruence rate in the analysis.

*Response: We have checked the points you mentioned and made the suggested corrections.*

*Actual place of death: To clarify this process, we have added the following text to the revised methods (lines 134–137): “The questionnaires were sent by the medical institutions that participated in the study and certified each patient's death. By linking the data obtained from medical institutions with the returned questionnaires, we were able to identify the place of death.”*

*We calculated both the congruence between the patient’s preferred place of death and the actual place of death, and the congruence between the caregiver's preferred place of death and the actual place of death as reported by caregivers.*

21. Which congruence measure was used in the analysis of factors associated with this outcome? Was it the congruence between the patient’s preferred and actual place of death, or the congruence between the bereaved caregiver’s preferred place of death for their loved one and the actual place of death? In the discussion section, the study refers to the congruence between the patient’s preferred and actual place of death. Please ensure consistency throughout the manuscript regarding which type of congruence was used in the analysis.

*Response: The section on “Congruence between preferred and actual place of death” in the “Questionnaires” has been revised to make it easier for readers to understand (lines 125–139).*

*For each case, the patient's preferred place of death (reported by the caregiver) was compared with the actual place of death to determine whether there was a match, and the number of patients for whom there was a match was calculated.*

22. Lines 222-226: Please rewrite these sentences for better clarity and review which factors show significant differences in Table 3.

*Response: Thank you; we have made the suggested corrections.*

23. In Table 3, which coefficient is used for the regression between the standardized and unstandardized coefficients? Please clarify.

*Response: We calculated partial regression coefficients, which are unstandardized coefficients. In Table 3, we have used "Partial Regression coefficient."*

24. Please consider discussing which factors affect the congruence between the preferred and actual place of death.

*Response: We have mentioned the factors that affect the congruence between the preferred and actual place of death in the revised discussion.*

25. I don't know whether this study measures quality of life. Be mindful not to overstate the findings of the study. Please align the discussion with the results (the dependent variable is the quality of dying).

*Response: Thank you; we have made the suggested corrections.*

26. Are you considering future research aimed at increasing the congruence rate between patients’ preferred and actual place of death?

*Response: Although we do not currently have any specific plans, we would like to conduct future research aimed at increasing congruence between the preferred and actual place of death.*
